# A Novel Motion Recognition Method Based on Force Myography of Dynamic Muscle Contractions

**DOI:** 10.3389/fnins.2021.783539

**Published:** 2022-01-13

**Authors:** Xiangxin Li, Yue Zheng, Yan Liu, Lan Tian, Peng Fang, Jianglang Cao, Guanglin Li

**Affiliations:** ^1^CAS Key Laboratory of Human-Machine Intelligence-Synergy Systems, Shenzhen Institutes of Advanced Technology (SIAT), Chinese Academy of Sciences, Shenzhen, China; ^2^Shenzhen Institute of Artificial Intelligence and Robotics for Society, Shenzhen, China; ^3^Shenzhen College of Advanced Technology, University of Chinese Academy of Sciences, Shenzhen, China; ^4^Shenzhen Engineering Laboratory of Neural Rehabilitation Technology, Shenzhen, China

**Keywords:** motion recognition, the rate of stress change, transient force-myography, piezoelectret, multifunctional prostheses

## Abstract

Surface electromyogram-based pattern recognition (sEMG-PR) has been considered as the most promising method to control multifunctional prostheses for decades. However, the commercial applications of sEMG-PR in prosthetic control is still limited due to the ambient noise and impedance variation between electrodes and skin surface. In order to reduce these issues, a force-myography-based pattern recognition method was proposed. In this method, a type of polymer-based flexible film sensors, the piezoelectrets, were used to record the rate of stress change (RSC) signals on the muscle surface of eight able-bodied subjects for six hand motions. Thirteen time domain features and four classification algorithms of linear discriminant analysis (LDA), K-nearest neighbor (KNN), artificial neural network (ANN), and support vector machine (SVM) were adopted to decode the RSC signals of different motion classes. In addition, the optimal feature set, classifier, and analysis window length were investigated systematically. Results showed that the average classification accuracy was 95.5 ± 2.2% by using the feature combination of root mean square (RMS) and waveform length (WL) for the classifier of KNN, and the analysis window length of 300 ms was found to obtain the best classification performance. Moreover, the robustness of the proposed method was investigated, and the classification accuracies were observed above 90% even when the white noise ratio increased to 50%. The work of this study demonstrated the effectiveness of RSC-based pattern recognition method for motion classification, and it would provide an alternative approach for the control of multifunctional prostheses.

## Introduction

Many people have been suffering from physical disabilities due to diseases, accidents, and natural disasters. For the people who have upper limb amputations, the loss of hand function causes a lot of inconvenience to the daily life. Therefore, a dexterous prosthesis is urgently needed to restore the functions of lost limbs for upper limb amputees. In a dexterous upper limb prosthesis, a reliable motion recognition system (MRS) that can accurately and quickly predict human’s motion intention is critical, since the actuator of a prosthesis performs according to commands outputted by the MRS.

Surface electromyogram (sEMG) is an electrophysiological signal generated by the contraction of muscles and can be non-invasively recorded with electrodes placed on the skin surface. Since sEMG contains rich human motion information, user’s motion intention would be recognized by decoding the sEMG patterns of different motion classes. Through this sEMG-based pattern recognition (sEMG-PR) approach, users can control the prostheses intuitively. Recently, the sEMG-PR method has been investigated in many laboratories all over the world (Castellini et al., 2014; Farina et al., 2014; Hahne et al., 2018; Guoying et al., 2021), and lots of research showed that the sEMG-PR method could achieve good classification performance for user’s motion intention (Zheng et al., 2014; Zheng and Wang, 2016; Zhuang et al., 2019). However, sEMG-PR-based motion recognition systems still has not yet realized clinical application because it encounters many problems in practical application. First, as a kind of electrophysiological signal, sEMG is weak and easily affected by movement artifacts and electromagnetic interferences of the surrounding environment (Fang et al., 2018). Second, sEMG electrodes are required to be well-contacted to the skin surface over the targeted muscles; however, issues of sweating skin (Zheng et al., 2014), failure of electrical conductive adhesive (Isakov et al., 1996; Dirks et al., 2018), and shifting of electrodes (Young et al., 2012; Fouad et al., 2015; Pan et al., 2015) will lead to impendence changes between electrodes and the skin surface, which make the sEMG signal acquisition instable. These problems could seriously affect the stability and reliability of the sEMG-PR method. Therefore, to find a new control signal source and develop a more reliable and stable motion recognition method are of great importance to improve the application performance of the intelligent prosthesis.

Force myography (FMG) that is generated by the muscle contractions when performing a movement can be recorded by placing a pressure sensor on the skin surface. Compared with sEMG, FMG is a kind of mechanical signal that is able to represent the pressure distribution in the radial direction of the skin surface over the target muscle, which is not affected by the change in impedance between the pressure sensor and the skin surface and is also robust to electromagnetic interferences. Considering the advantages of FMG signal, some researchers proposed to use FMG signals for motion recognition. Curcie et al. (2001) used eight myo-pneumatic sensors to capture pressure signals on the surface of the residual forelimb and develop a linear filter that can decode three specific finger flexion commands. Radmand et al. (2016) proposed a technique of high-density FMG to distinguish the pressure patterns. In this technique, 126 force-sensitive resistors were used, and the classification accuracy of eight hand and wrist movements is over 99%. Ahmadizadeh et al. (2017) compared the performance of FMG and EMG in hand gesture classification and showed that the FMG signals achieved the higher classification accuracy of 96.7% (Jiang et al., 2017). Belyea et al. (2019) examined the usability of FMG for real-time prosthesis control and demonstrated that the FMG-based scheme outperformed the EMG-based scheme in both sequential classification and simultaneous regression control. These results showed the feasibility of FMG to be an alternative or synergist to EMG for motion recognition. However, the FMG signals in most of these studies were acquired by using the force-sensing resistors (FSRs) that converts the pressure distributions at skin surface to the impedance of the resistors. The main drawbacks of FSR are the low resolution and slow response speed to muscle contractions, which would delay the response time of prosthesis in clinical application.

In our previous study (Fang et al., 2018), a type of novel force-sensitive sensor with high sensitivity to muscle deformation was developed. This sensor is made of space-charge piezoelectret films that can generate voltage in response to the rate of stress change (RSC); therefore, we can use the sensors to detect dynamic pressure distributions on the skin surface. On the basis of the primary results in Fang et al. (2018), a novel motion recognition method based on RSC signals was proposed in this study. The RSC signals of six hand and wrist motion classes were recorded by using eight piezoelectret sensor sensors placed on the forearm of subjects, and then, 13 time domain (TD) features were extracted from the RSC signals and four algorithms of linear discriminant analysis (LDA), K-nearest neighbor (KNN), artificial neural network (ANN), and support vector machine (SVM) were adopted to decode the RSC signals of different motion classes. By comparing the classification performances, the optimal feature set, classifier, and analysis window length were selected. Additionally, the robustness of the proposed RSC-based method against white noise was also investigated. The work of this study may provide a promising alternative approach for motion pattern recognition.

## Materials and Methods

### Subjects

Eight able-bodied (AB) subjects (three female and five male) were recruited for the experiments conducted in this study. The subjects were all right-hand dominated and aged from 21 to 24 years old. The arm circumferences of eight subjects were ranged from 22.8 to 28.6 cm, with the average value of 25.2 ± 2.2 cm. The experimental protocol was approved by the Institutional Review Board of Shenzhen Institute of Advanced Technology, Chinese Academy of Sciences. All subjects have given written informed consent and provided permission for the publication of photographs for scientific and educational purpose.

### Force Myography Sensors

**FIGURE 1:**
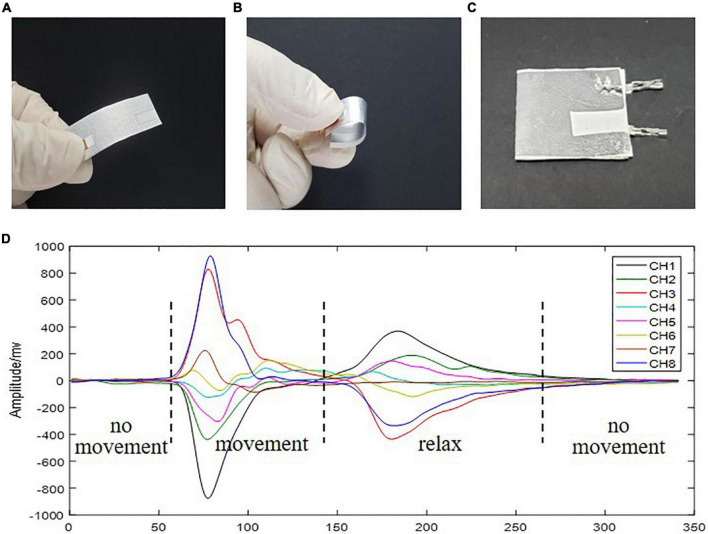
**(A,B)** A flexible space-charge piezoelectret film sample, **(C)** a packaged piezoelectret sensor for pressure measurement, and **(D)** an example of multichannel RSC signals measurement by the piezoelectret sensors.

**FIGURE 2:**
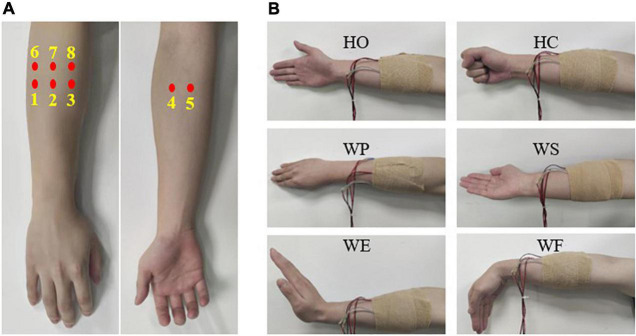
**(A)** positions of sensor placement, **(B)** motion classes involved in this study.

In this work, force-sensitive sensors were prepared from piezoelectret films that can generate voltage in response to rate of stress change (RSC) on its normal direction. The piezoelectrets have properties of small thickness, light weight, flexibility, stretchability, and high sensitivity, making them very suitable for stress change measurement in wearable devices (Ma et al., 2017; Chen et al., 2019; Fang et al., 2021), as shown in Figures 1A,B. Each packaged sensor unit was 1.2 × 1.2 cm^2^ in area and 0.24 mm in thickness, as shown in Figure 1C. An example of RSC signal acquire by the piezoelectret sensor was presented in Figure 1D, in which the piezoelectret sensor showed a good response to the dynamic stages of a movement. Eight piezoelectret sensors were used to record the RSC signals produced by muscle contractions. For each subject, the eight sensors were placed on the forearm of user’s dominant hand: sensors 1–6 were placed over the radial aspect of the main extensor bundle, while sensors 7 and 8 were placed on the ulnar aspect flexor bundle, determined by the palpation. All of the sensors were fastened with a bandage, as shown in Figure 2.

### Signal Acquisition

Six motion classes of hand close (HC), hand open (HO), wrist pronation (WP), wrist supination (WS), wrist extension (WE), and wrist flexion (WF) were tested, as shown in Figure 2B. Prior to the FMG recordings, each subject had a 10-min practice time to get familiar with the motions and the experimental procedures. During the experiment, each subject sat on a chair in a comfortable manner and was asked to follow demonstrations of each motion class displayed on the computer screen in front of them. Since piezoelectret sensors detect the pressure variation produced by muscle contraction, the RSC signal is zero when muscle contractions hold at a consistent effort level. Therefore, each subject was requested to perform a motion when the picture appeared on the computer screen and finish the motion within 2 s without holding muscle contraction for a while. Each motion was repeated 30 times, and there was a short rest of 2 s between each two repetitions and a long rest of 2 min after every 10 repeats to avoid muscle fatigue. The RSC signals were collected by a data-acquisition card (USB-0816) with a sampling rate of 100 Hz and then transmitted to a computer for further processing.

### The Rate of Stress Change Based Pattern Recognition Method

#### Data Preprocessing

**FIGURE 3:**
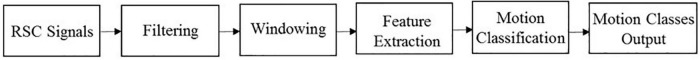
Procedure of RSC-based motion pattern recognition.

The RSC-based motion pattern recognition (RSC-MPR) procedure is shown in Figure 3. The acquired RSC signals were processed offline by using Matlab@2010b. A 50-Hz notch filter was applied to remove the power-line interference prior to further preprocessing of the RSC signals. Then, the filtered RSC signals from 30 replications were concatenated to form an analysis set for each class of motions. The concatenated RSC signal sets were segmented using a sliding analysis window with a length of 300 ms and an increment of 100 ms, since it was demonstrated to achieve the better classification performance in our preliminary experiment. From each analysis window, signal features were extracted to train and test a classifier.

#### Feature Extraction

An appropriate feature representation is important for motion pattern recognition. In order to select the optimal RSC features, 13 frequently used time domain (TD) features with high computational efficiency for motion recognition were evaluated in this study (Tkach et al., 2010; Phinyomark et al., 2013; Vidovic et al., 2016).

(1) Mean absolute value

Mean absolute value (MAV) is an average of absolute value of the RSC signal *x* in an analysis window with N samples, as calculated in Eq. (1).


(1) $$MAV=\frac{1}{N}\sum_{i=1}^{N}\left|x_{i}\right|$$

(2) Waveform length

Waveform length (WL) is the cumulative length of the RSC signal in the analysis window and is calculated in Eq. (2)


(2) $$WL=\sum_{i=1}^{N-1}\left|x_{i+1}-x_{i}\right|$$

(3) Average amplitude change

Average amplitude change (AAC) is the mean value of WL, which is a measure to evaluate the complexity of RSC signal x, and formulated as


(3) $$AAC=\frac{1}{N}\sum_{i=1}^{N-1}\left|x_{i+1}-x_{i}\right|$$

(4) Simple square integral

Simple square integral (SSI) is a summation of square value of the RSC signal amplitude. It is usually used as an energy index for a signal and defined as Eq. (4).


(4) $$SSI=\sum_{i=1}^{N}x_{i}^{2}$$

(5) Root mean square

Root mean square (RMS) is the measure of power for the RSC signal x. The computation is in Eq. (5), in which N is the length of an analysis window, and x_*i*_ is the *i*th sample in the analysis window.


(5) $$\text{RMS}=\sqrt{\frac{1}{N}\sum_{i=1}^{N}x_{i}^{2}}$$

(6) Variance

Variance (VAR) of RSC is defined as an average of square value of the deviation of RSC signal x in an analysis window with N samples.


(6) $$VAR=\frac{1}{N-1}\sum_{i=1}^{N}{\left(x_{i}-\frac{1}{N}\sum_{i=1}^{N}x_{i}\right)}^{2}$$

(7) Standard deviation

Standard deviation (STD) is the square root of VAR. It is an index of dispersion between individuals within the RSC signal x in an analysis window with N samples.


(7) $$STD=\sqrt{\frac{1}{N-1}\sum_{i=1}^{N}{\left(x_{i}-\frac{1}{N}\sum_{i=1}^{N}x_{i}\right)}^{2}}$$

(8) Difference absolute standard deviation value

Difference absolute standard deviation value (DASDV) is a standard deviation value of the wavelength, and it is defined by(8)


(8) $$DASDV=\sqrt{\frac{1}{N-1}\sum_{i=1}^{N-1}{\left(x_{i+1}-x_{i}\right)}^{2}}$$

(9–11) Absolute value of the third, fourth, and fifth temporal moment

Temporal moment is a statistical analysis that was used to reduce the within class separation for the odd moment case, the third, fourth, and fifth temporal moment (TM3, TM4, and TM5) are defined as:


(9) $$TM3=\left|\frac{1}{N}\sum_{i=1}^{N}x_{i}^{3}\right|$$

(10) $$TM4=\left|\frac{1}{N}\sum_{i=1}^{N}x_{i}^{4}\right|$$

(11) $$TM5=\left|\frac{1}{N}\sum_{i=1}^{N}x_{i}^{5}\right|$$

(12) Kurtosis

Kurtosis (KURT) is the normalized fourth-order central moment, which is a measure of the varying speed of the RSC signals and calculated as:


(12) $$KURT=\frac{\frac{1}{N}\sum_{i=1}^{N}{\left(\left|x_{i}\right|-\frac{1}{N}\sum_{i=1}^{N}x_{i}\right)}^{2}}{{\left(\sqrt{\frac{1}{N}\sum_{i=1}^{N}x_{i}^{2}}\right)}^{4}}$$

(13) Log detector

The same as in EMG application, log detector (LOGD) also can be considered as an estimate of the muscle contraction force for RSC signals, and it is defined as Eq. (13).


(13) $$LOGD=e^{\frac{1}{N}\sum_{i=1}^{N}log\left|x_{i}\right|}$$

#### Motion Classification

After feature extraction, four classification algorithms of LDA, KNN, ANN, and SVM were applied for classifying RSC patterns of different motions. These four algorithms were proposed in many previous researches and widely used for limb motion intent decoding. In this study, the parameter K was set as 3 for the algorithm of KNN. The classifier of ANN has two hidden layers with 16 and 6 neurons, respectively. In the classifier of SVM, the kernel function was radial basis function, and the one-against-one (1-v-1) strategy was used to extend the two-class SVM to a multiclass SVM. In the 1-v-1 strategy, a SVM classifier was trained for each two motion classes and a total of N × (N - 1)/2 classifiers were trained for N classes. Thus, there are N × (N - 1)/2 classification outputs for each feature sample, and the output with the greatest number of occurrences is chosen as the final classification result.

### Performance Evaluation

In order to evaluate the performance of the 13 TD features and four classifiers, the metric of classification accuracy (CA) was adopted in this study, as expressed in Eq. (14). The average CAs of the six motion classes were obtained by using the method of fourfold cross-validation for each subject.


(14) $$CA=\frac{Numberofcorrectlyclassifiedsamples}{Totalnumberoftestingsamples}\times 100\%$$

Additionally, the sequence forward search (SFS) algorithm, which was referenced in Tkach et al. (2010), was used to investigate the effect of number of features on the classification performance to select an optimal time domain feature (OTDF) combination.

Furthermore, in order to investigate feature stability with respect to white noise that is unavoidably caused by electronic circuit, we added white Gaussian noise into the raw RSC signals with signal-to-noise ratio (SNR) of 20, 40, 60, 80, and 100%. Then, the TD features were extracted from the contaminated RSC signals and used for classification of the six motion classes.

## Results

### Rate of Stress Change Signals

**FIGURE 4:**
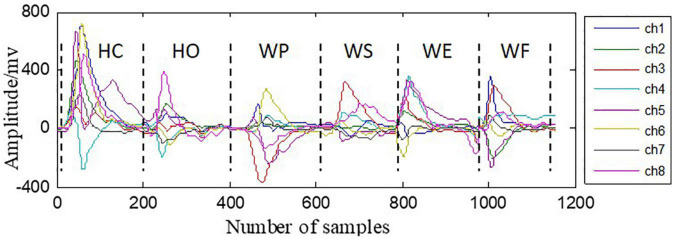
An example of the RSC signals of the eight channels and six hand motions.

An example of the eight-channel RSC signals for six hand motions is illustrated in Figure 4. From the figure, it can be observed that shapes of RSC signals were distinguishable between the six motion classes with respect to amplitudes, shape, and directions.

### Feature Set and Classifier Selection

The average classification accuracies and standard deviations obtained for the 13 TD features across subjects were ranked in decreasing order based on the LDA classification results, as shown in Table 1. It can be seen that the feature of RMS achieved the best classification accuracies of 83.5 ± 3.4%, 91.7 ± 2.5%, 90.5 ± 3.2%, and 92.1 ± 2.1% for LDA, KNN, ANN, and SVM, respectively. The feature of MAV ranked second with classification accuracies about 1% lower than those of RMS. Additionally, compared with the classification algorithms of KNN, ANN, and SVM, LDA achieved the lowest average classification accuracy of 58.7% and the highest standard deviation of 6.2% across the 13 TDFs.

**TABLE 1:** Average motion classification accuracy of the 13 time domain features (TDFs) across all the subjects and motion classes by using LDA, KNN, ANN, and SVM, respectively.

**Rank**	**Feature**	**LDA (%)**		**KNN (%)**		**ANN (%)**		**SVM (%)**	
		**MEAN**	**SD**	**MEAN**	**SD**	**MEAN**	**SD**	**MEAN**	**SD**
1	RMS	83.5	3.4	91.7	2.5	90.5	3.2	92.1	2.1
2	MAV	82.3	3.4	90.4	2.5	89.1	3.0	91.0	2.1
3	LOGD	77.9	3.8	86.6	2.9	85.3	3.9	86.8	2.6
4	SSI	67.4	6.6	87.0	3.0	88.9	3.3	82.2	2.6
5	LEN	66.0	7.8	79.9	4.2	79.2	5.9	76.8	6.7
6	AAC	65.8	7.8	80.1	4.2	79.1	5.7	76.8	7.0
7	STD	63.4	7.1	75.8	5.2	75.0	5.7	73.3	6.8
8	DASDV	62.0	4.4	81.2	4.0	76.8	6.7	80.1	4.8
9	VAR	50.8	8.3	69.1	4.7	71.9	6.5	56.2	8.1
10	TM3	49.0	9.3	80.0	3.7	84.3	3.4	70.5	2.6
11	TM4	40.7	9.1	80.0	3.1	81.4	8.1	67.5	2.9
12	TM5	34.9	7.5	75.6	3.6	74.1	7.2	61.8	2.9
13	KURT	19.0	2.2	25.0	3.4	18.4	1.8	23.8	4.0
	Average	58.7	6.2	77.1	3.6	76.5	5.0	72.2	4.2

**FIGURE 5:**
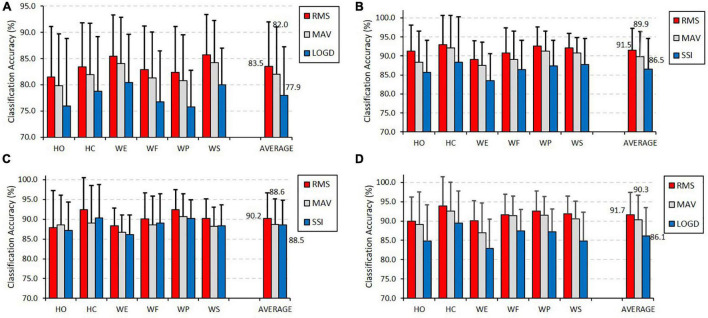
Classification performance of the six motion classes achieved by the top 3 individual optimal TDFs for classifiers of (A) LDA, (B) KNN, (C) ANN, and (D) SVM.

Figure 5 depicts the classification performance of the six motion classes achieved by the top 3 optimal TDFs, which are marked in gray color in Table 1 for the four different classifiers. It could be observed from Figure 5 that the feature of RMS obtained the highest classification accuracy for all the six motion classes no matter what classification algorithms was used. Especially, the classification accuracies of the six motion classes were all above 90% when using RMS in SVM. It was found that the motion class of HC had the highest classification accuracy above 92% among the six motion classes in KNN, ANN, and SVM; however, it had the lowest classification accuracy of 81.6% in LDA. Similarly, the motion of WE that had the lowest classification accuracy when using KNN, ANN, and SVM obtained the highest classification accuracy in LDA.

**FIGURE 6:**
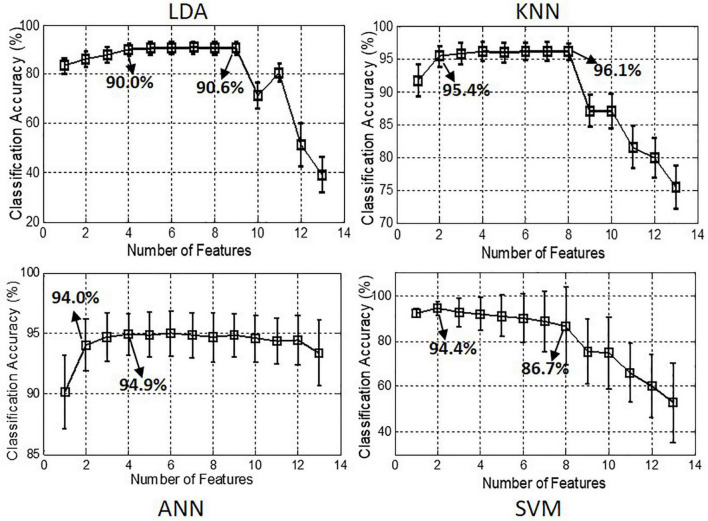
Classification accuracies achieved by using different number of features for classifiers of LDA, KNN, ANN, and SVM, respectively.

Figure 6 demonstrates the classification accuracies achieved by using different numbers of TD features. It can be seen that the classification performance improved slightly when using more TDFs. For classifiers of LDA and ANN, the classification accuracies increased about 5% when the number of features increased from 1 to 4 and did not further improve when the number increased more. Even when the number of features increased more than nine, the classification accuracy was greatly decreased, e.g., from 90.6 to 40% for LDA.

Table 2 shows the optimal TD feature set with the number of features ranged from 1 to 4. For LDA, the optimal feature set consisted of RMS, STD, VAR, and WL, achieving the classification accuracy of 90.0 ± 2.6%. For KNN, the optimal feature set consisted of RMS and WL, which had the fewer number of features but obtained the higher classification accuracy of 95.5 ± 2.2%. For ANN, the optimal feature combination included RMS, ACC, VAR, and SSI, with the classification accuracy of 95.0 ± 1.9%. For SVM, the highest classification accuracy was 94.4 ± 2.3% and achieved by the optimal feature set of RMS and STD.

**TABLE 2:** Classification performance (averaged CA ± SD %) for the optimal time domain feature (OTDF) combinations with the number of features from 1 to 4.

	**OTDF_1**	**OTDF_2**	**OTDF_3**	**OTDF_4**

**LDA**	**(RMS)**	**(RMS STD)**	**(RMS STD VAR)**	**(RMS STD VAR WL)**

	83.5 ± 3.4	86.4 ± 3.3	88.1 ± 3.1	**90.0 ± 2.6**

**KNN**	**(RMS)**	**(RMS WL)**	**(RMS WL STD)**	**(RMS WL STD KURT)**

	91.7 ± 2.8	**95.5 ± 2.2**	95.8 ± 2.4	96.1 ± 2.2

**ANN**	**(RMS)**	**(RMS AAC)**	**(RMS AAC VAR)**	**(RMS AAC VAR SSI)**

	90.2 ± 3.3	93.9 ± 3.1	94.5 ± 3.5	**95.0 ± 1.9**

**SVM**	**(RMS)**	**(RMS STD)**	**(RMS STD KURT)**	**(RMS STD KURT DASDV)**

	92.5 ± 2.4	**94.4 ± 2.3**	92.8 ± 2.7	92.1 ± 1.5

*The feature combination with the classification accuracies showed in bold values was the optimal feature combination for each classifier.*

### Effect of Analysis Window Length

**FIGURE 7:**
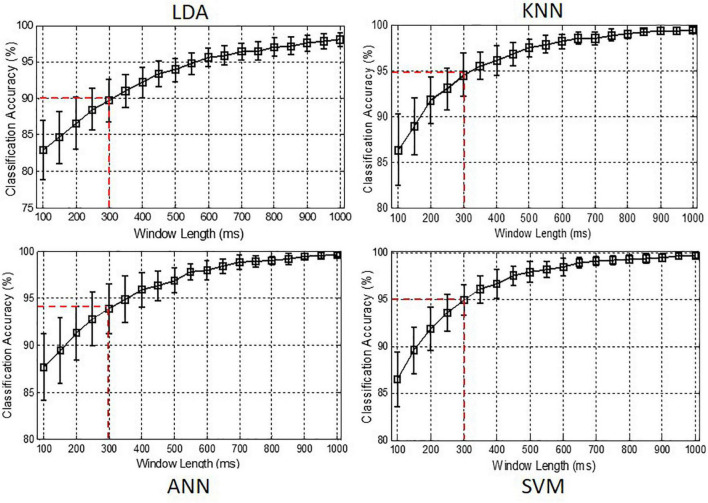
Classification accuracies achieved by using different window lengths for classifiers of LDA, KNN, ANN, and SVM, respectively.

Figure 7 shows the effect of analysis window length on motion classification performance. It can be seen that the better classification performance was obtained by using the larger window length. Especially, the classification accuracies increased rapidly when the window length increased from 100 to 300 ms. For the classifiers of KNN and SVM, the classification accuracies were increased to about 95% when the window length was 300 ms. Considering that the larger window length will yield larger computational load and longer response time, 300 ms was selected as the optimal analysis window length.

### Overall Recognition Performance of Rate of Stress Change-Based Motion Pattern Recognition Method

**FIGURE 8:**
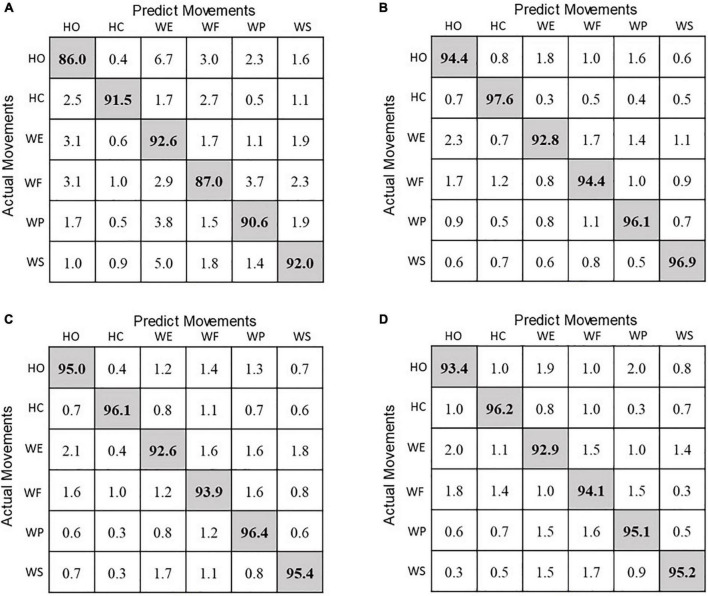
Confusion matrix of classification accuracies (%) for **(A)** LDA, **(B)** KNN, **(C)** ANN, and **(D)** SVM classifiers.

Based on the parameters determined aforesaid (optimal feature set and 300-ms window length), the confusion matrix of classification accuracies of the six motion classes obtained by different classifiers is shown in Figure 8. It was observed that LDA achieved the worst classification performance for each motion class compared with other classifiers. The classification accuracies of the six motion classes achieved by KNN were slightly higher than those of others. Additionally, it was found that the motion classes of HO, WE, and WF had the lower accuracies, and HC, WP, and WS had the higher accuracies above 95% for of the classifiers of KNN, ANN, and SVM classifiers.

### Robustness Performance of Rate of Stress Change-Based Motion Pattern Recognition Method for White Noise

**FIGURE 9:**
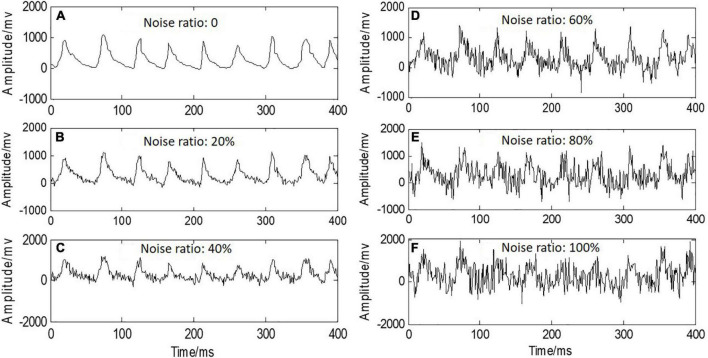
An example of RSC signals added with whiten Gaussian noise with noise ratio from 0 to 100%.

**FIGURE 10:**
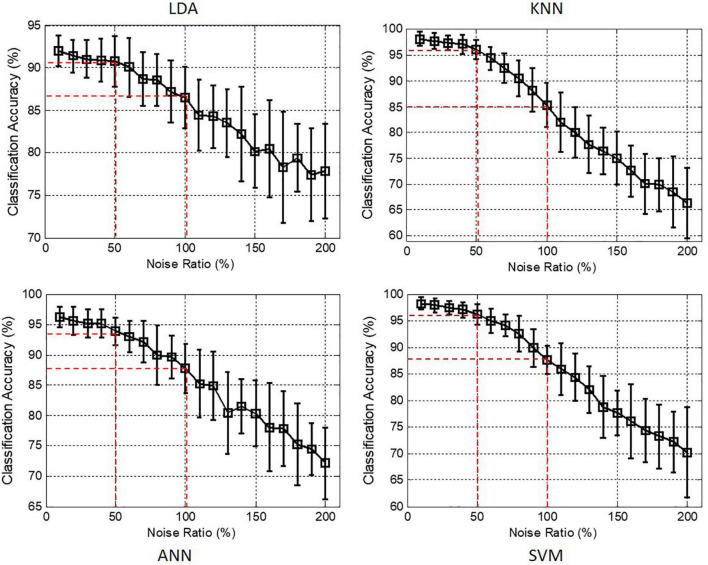
Classification accuracies achieved by the optimal feature combinations for LDA, KNN, ANN, and SVM classifiers when RSC signals were contaminated with whiten noise.

In order to investigate the robustness of RSC in motion recognition, whiten Gaussian noise with different noise ratios of 0, 20, 40, 60, 80, and 100% was added into the raw RSC signals, and the contaminated RSC signals are shown in Figure 9. It was observed that distortions of RSC signals were caused by whiten noise with the higher noise ratio of 80 and 100%. Figure 10 shows the classification accuracies achieved by using the optimal feature set of the four classifiers when RSC signals were contaminated with whiten noise. It can be seen that there was almost no effect on the classification performance when the noise ratio increased to 50%, with the classification accuracies above 95% for both of KNN and SVM. When the noise ratio increased to 100%, the classification accuracies were still above 85% for all of the four classifiers.

## Discussion

A sensitive and accurate motion recognition method is crucial for multifunctional prostheses. Even though the sEMG-based motion recognition has been researched in many laboratories over the world and obtained lots of achievements (Castellini et al., 2014; Farina et al., 2014; Vidovic et al., 2016; Li et al., 2017), a large-scale commercial application in multifunctional prostheses is still not realized. One of the important issues is the poor robustness against external electromagnetic interferences, skin sweating, and electrodes shifts (Biddiss and Chau, 2007). In this study, a novel RSC-based motion recognition method that is capable of detecting dynamic pressure distributions on the skin surface was proposed.

Muscle contraction is a dynamic process, consisting of the movement start, movement duration, and movement end. However, in most motion recognition methods, the sEMG signals or FMG signals were acquired from the stage of movement duration, i.e., during muscle contractions, which is a static contraction and would lead to high classification error rates in the dynamic stages of movement start and end (Scheme et al., 2013; Simon et al., 2011). From another point of view, a sensor that is able to capture signals precisely in dynamic stages is very critical to extract dynamic information for muscle contractions. Different from some other types of pressure sensors like resistive or capacitive sensors, the piezoelectret (PET) sensors used in this study can generate output in response to only pressure change, and there is no output by a static pressure. This characteristic makes piezoelectret sensors very suitable for dynamic measurement, i.e., they have a good dynamic response in pressure sensing. What is more, the material properties of piezoelectrets such as film-like, flexible, and highly sensitive make them competitive in wearable applications like human body signal acquisition. The results of this study have confirmed the promising performances of piezoelectret sensors in detecting RSC that is in the normal direction of the sensor/skin interface, and the faster is the muscle deformation, the greater is the RSC amplitude. Furthermore, it can be observed from Figure 4 that the shapes of RSC signals are distinguishable with respect to directions and amplitudes for different motion classes and electrodes. Therefore, it can be demonstrated that the proposed RSC-based method is able to recognize the dynamic movement patterns without subjects holding contractions for a while. It is promising for amputees to control the RSC-based prosthetic systems in a natural manner like the way humans engage in the activities of daily living.

Appropriate features and effective classification algorithms are two crucial factors for motion recognition (Simon et al., 2011). To select the optimal RSC features and classification algorithms, 13 TD features and four algorithms of LDA, KNN, ANN, and SVM were investigated in this study. It can be seen from Table 1 that no matter what classification algorithm was used, the feature of RMS obtained the best classification performance among the 13 TD features. This result confirms the conclusion that an appropriate feature representation is more important than the choice of classification algorithms for motion recognition (Phinyomark et al., 2008; Adewuyi et al., 2016; Kamavuako et al., 2016).

It is known to all that using a feature set with two or more combined features is an effective approach to improve pattern classification performance (Ma et al., 2017). However, it was shown that the classification performance was not always improved as the number of feature increased (Figure 6). Especially for the classifiers of LDA, KNN, and SVM, the classification accuracies were dramatically reduced when the number of feature was increased more than nine. This demonstrated that there was much redundant information between these features, which had negative effects on the classification performance. Therefore, the optimal number of features should be determined first in the motion intention recognition. As presented in Table 2, it can be seen that the optimal number and combination of the features were different when applied in different classification algorithms. It is noted that for the classifier of KNN, the classification accuracy achieved by the feature combination of (RMS and WL) was 95.5 ± 2.2%, and the classification accuracy achieved by the feature combination consisted of four features (RMS, WL, STD, and KURT) was 96.1 ± 2.2%, which was only 0.6% increased. Considering that using more number of features will increase the computational complexity, RMS and WL were selected as the optimal feature combination for KNN. Therefore, the optimal classification performances were achieved by the feature combinations of (RMS and WL) and (RMS, ACC, VAR, and SSI) for the classifiers of KNN and ANN, respectively, with the classification accuracies were above 95%.

Rapid response is an important issue in real-time prosthesis control. In Figure 7, the classification accuracy was observed to be improved about 15% when the analysis window length increased from 100 to 1,000 ms. This is because the larger window contains more RSC information of user’s movements. However, the larger analysis window length also means heavier computational load, which will lead to a longer response time for prosthesis control. Note that using a 300-ms analysis window achieved the classification accuracies about 95% for KNN and SVM (Figure 7) and could save huge computation loads compared to using a 1,000-ms analysis window. As the conclusion conducted in previous studies, a response delay more than 300 ms will be perceived by prosthesis users (Englehart and Hudgins, 2003). Thus, 300 ms was selected as the optimal analysis window length in this study. With the optimal parameters, the classification accuracies of the six motion classes were all above 92.0% for KNN, ANN, and SVM as shown in Figure 8, which is comparable to the motion recognition performance of sEMG-based method (Biddiss and Chau, 2007; Simon et al., 2011; Scheme et al., 2013).

Furthermore, the robustness of RSC-based motion recognition method to white noise was investigated. Unlike some human physiological signals, e.g., electroencephalogram (EEG), that were susceptible to external noise interference, RSC signals showed a strong robustness against white noise, and the RSC-based motion recognition method achieved good classification performance when the white noise ratio increased to 50% (Figure 10). Even when the noise ratio increased to 100%, the classification accuracies achieved by the four classifiers were still above 80%. Interestingly, it was found that even though the classifier of KNN achieved the best classification performance among all the four classifiers, its robustness against white noise was the poorest. Contrarily, the classifier of LDA, which obtained the lowest classification accuracy, had the strongest robustness.

Although the proposed RSC-based method achieved the satisfactory results in motion intention recognition, its classification performance was not compared to that of the sEMG-based method, which should be conducted in future works. Moreover, the study of a multisensor confusion method that combined sensors of PET and FSR together would be conducted in future work to record the signals of static and dynamic muscle contractions simultaneously, which would contain more muscle information and might further improve the motion recognition performance. Additionally, amputees will be recruited, and the real-time performance of RSC-based method should be investigated in the multifunctional prosthesis control.

## Conclusion

This study proposed a novel RSC-based motion recognition method. In this method, a novel piezoelectret force-sensitive sensor was used to record the RSC signals that were produced by dynamic pressure distributions on the skin surface. By comparing the performance of 13 time domain features and four classification algorithms, the optimal classification performances were 95.5 ± 2.2% and 95.0 ± 1.9% achieved by the feature combinations of (RMS and WL) and (RMS, ACC, VAR, and SSI) for the classifiers of KNN and ANN, respectively, with a 300-ms analysis window length. Additionally, by investigating the effect of white noise on the classification performance, the RSC-based motion recognition method showed a strong robustness to white noise. Therefore, the work of this study would provide help to improve the robustness of motion pattern recognition, and the proposed RSC-based method may be a promising alternative approach for multifunctional prosthesis control.

## Data Availability Statement

The raw data supporting the conclusions of this article will be made available by the authors, without undue reservation.

## Ethics Statement

The studies involving human participants were reviewed and approved by the Institutional Review Board of Shenzhen Institute of Advanced Technology, Chinese Academy of Sciences. The patients/participants provided their written informed consent to participate in this study.

## Author Contributions

XL, PF, and GL designed the research. XL and YZ performed the research. YL, LT, and JC contributed to the data acquisition software and the piezoelectret sensors. XL wrote the manuscript. PF and GL contributed to the funding acquisition and writing—review and editing. All authors contributed to the article and approved the submitted version.

## Conflict of Interest

The authors declare that the research was conducted in the absence of any commercial or financial relationships that could be construed as a potential conflict of interest.

## Publisher’s Note

All claims expressed in this article are solely those of the authors and do not necessarily represent those of their affiliated organizations, or those of the publisher, the editors and the reviewers. Any product that may be evaluated in this article, or claim that may be made by its manufacturer, is not guaranteed or endorsed by the publisher.
